# Hyaluronic Acid–Zein Core-Shell Nanoparticles Improve the Anticancer Effect of Curcumin Alone or in Combination with Oxaliplatin against Colorectal Cancer via CD44-Mediated Cellular Uptake

**DOI:** 10.3390/molecules27051498

**Published:** 2022-02-23

**Authors:** Lu Liu, Shufang Yang, Feng Chen, Ka-Wing Cheng

**Affiliations:** 1 Institute for Food and Bioresource Engineering, College of Engineering, Peking University, Beijing 100871, China; liulugongxue@pku.edu.cn; 2 Shenzhen Key Laboratory of Marine Microbiome Engineering, Institute for Advanced Study, Shenzhen University, Shenzhen 518060, China; ysf_nbj@163.com; 3 Institute for Innovative Development of Food Industry, Shenzhen University, Shenzhen 518060, China

**Keywords:** curcumin, oxaliplatin, hyaluronic acid–zein composite nanoparticles, colorectal cancer, cellular uptake

## Abstract

Curcumin (CUR) has been reported to enhance the chemotherapeutic efficacy of oxaliplatin (OXA) in colorectal cancer (CRC) and inhibit OXA-induced side effects. However, shortcomings, including poor solubility and sensitivity to metabolic transformation, have greatly undermined its value in clinical applications. In this study, the potential of CUR-encapsulated hyaluronic acid (HA)–zein composite nanoparticles (HZ-CUR) as an oral adjuvant for OXA-based chemotherapy was assessed in representative CRC models in mice. Cell viability and colony formation assays in three human CRC cell lines showed that HZ-CUR had a stronger anti-CRC effect than free CUR when given alone and a stronger synergistic effect when combined with OXA, especially in HCT116 and HT29 cell lines. Western blotting, cellular uptake, and RNA interference assays revealed that OXA-induced upregulation of CD44 likely contributed to enhanced cellular uptake of HZ-CUR and thus the enhanced anticancer effect. The significantly improved anti-CRC effects and potential underlying mechanism of HZ-CUR alone and in combination with OXA were further validated in a subcutaneous xenograft and an in situ CRC model in mice. These findings support that HZ-CUR may be an effective oral adjuvant for OXA-based CRC chemotherapy that would not only improve its efficacy but also help reduce the associated side effects.

## 1. Introduction

Colorectal cancer (CRC) continues to be the third most common and fatal cancer worldwide, with an increasing incidence [1]. Oxaliplatin (OXA) is one of the most commonly used first-line chemotherapeutic agents for CRC. However, its frequent use has been undermined by the occurrence of drug resistance and severe adverse events, such as hematologic toxicities, peripheral neuropathy, and hypersensitivity [2,3,4]. Curcumin (CUR), a bioactive flavonoid derived from turmeric, has been reported to enhance the chemotherapeutic efficacy of OXA by inducing apoptosis and inhibiting CXC-Chemokine/NF-κB and TGF-β/Smads signaling pathways in CRC cells [5,6,7]. It has also been reported to alleviate OXA-induced liver injury and peripheral neuropathic pain [8,9]. Phase II clinical data of metastatic CRC patients indicate that CUR is a safe and tolerable adjunct to folinic acid/5-fluorouracil/OXA (FOLFOX) chemotherapy [10]. However, poor solubility, pH sensitivity, and rapid metabolism of CUR result in its low bioavailability, thus limiting its value in clinical applications [11]. Various delivery strategies have been proposed to increase the bioavailability of CUR, and significant progress has been made in the development of CUR-encapsulated nanoformulations, which hold great promise for overcoming some of the above-mentioned limitations [12,13]. In this regard, biocompatible and biodegradable natural macromolecules, such as polysaccharides and proteins, have been widely used as key components of the formulations owing to their generally high safety profiles [14,15,16].

Zein, a major protein of corn, has been demonstrated to be a favorable material for encapsulating hydrophobic bioactive compounds due to its unique amphiphilic, self-assembling, and swelling properties [17,18]. In recent years, polysaccharide–zein nanoparticles have attracted a lot of attention as carriers of hydrophobic labile nutraceuticals. The formation of a polysaccharide coating layer could help to improve the physical and chemical stability of zein nanoparticles [19,20]. Moreover, the incorporation of polysaccharides has also been shown to endow polysaccharide–zein nanoparticles with specific functions, such as cancer cell targeting [21]. Hyaluronic acid (HA) is a glycosaminoglycan with a large number of hydroxyl and carboxyl groups. Its excellent water-binding capacity and biocompatibility make it a popular raw material for antiaging matrices, soft-tissue filler, and drug delivery [22]. HA has been proven to be capable of specific binding to a cell surface receptor CD44, which is overexpressed in various solid tumors and is a key cancer stem cell marker [23]. The specific recognition/interaction between HA and CD44 contributes to the tumor-targeting property of HA-coated nanocarriers [24]. In particular, a recent study showed that CD44 expression enhanced chemoresistance of CRC cells to OXA and fluorouracil and was an adverse prognostic factor for patients with colorectal liver metastases after chemotherapy [25]. Therefore, CD44 targeting is a sensible strategy for achieving better efficacy in OXA-based chemotherapy. In this context, HA may be a highly favorable polysaccharide for the coating of CUR-encapsulated zein nanoparticles to facilitate the targeted delivery of CUR to CRC cells that would ultimately contribute to enhanced therapeutic efficacy and reduced side effect of OXA.

Among the different routes of administration, oral delivery has been the most highly sought-after due to advantages such as convenience and good patient compliance. Our preliminary data showed that compared with free CUR, CUR-encapsulated HA–zein composite nanoparticles (HZ-CUR) exhibited significantly improved pharmacokinetics and tissue distribution behavior after oral administration in a subcutaneous CRC-xenograft model in mice [26]. These results suggest that HZ-CUR has promising potential as an efficient oral delivery system of CUR. The present study therefore aimed to evaluate the therapeutic efficacy of oral HZ-CUR nanoparticles both alone and in combination with OXA in representative CRC models (subcutaneous and in situ). Furthermore, the underlying mechanism was partially characterized, focusing on CD44-mediated cellular uptake of the test agents.

## 2. Results and Discussion

### 2.1. Characterization of Blank and CUR-Loaded Nanoparticles

**Figure 1:**
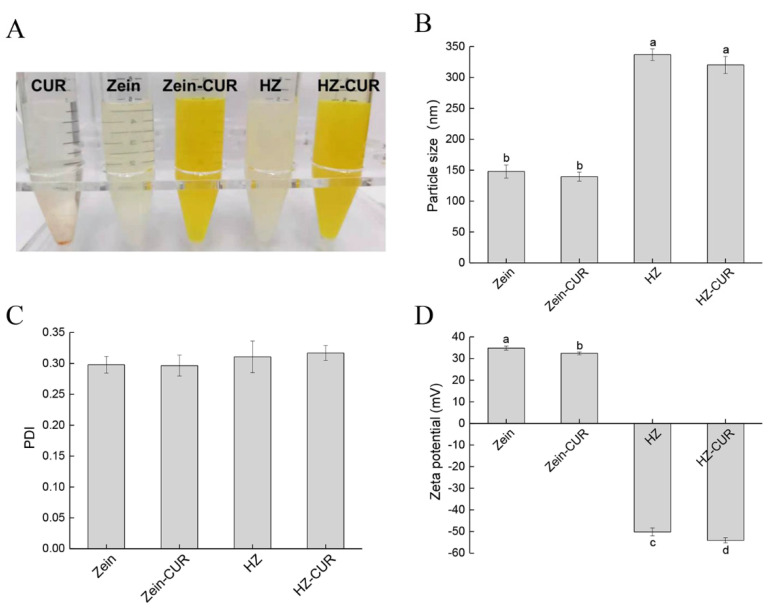
Characterization of composite nanoparticles. (**A**) Macro images, (**B**) particle size, (**C**) polydispersity index (PDI), and (**D**) zeta potential. Values are means ± SD, n = 3. Groups marked with different letters (a, b, c, d) have significant differences (*p* < 0.05).

As shown in Figure 1A, all the nanoparticles were evenly distributed in an aqueous solution. This was consistent with the field-emission scanning electron microscopy (FE-SEM) data in our recent study [26]. The encapsulation of zein visually improved the solubility of CUR, likely due to the high content of hydrophobic amino acid residues on zein molecules, which enable them to have high-affinity interaction with CUR [17]. Upon the addition of HA, the turbidity of the nanoparticles’ solution increased, which could be attributed to the increase in particle size (from 147.9 nm (Zein) to 337.0 nm (HZ)). CUR did not seem to have any significant effect on particle size (Figure 1B). Meanwhile, the similar PDI values of the nanoparticles indicated that HA did not significantly affect particle size distribution (Figure 1C). The cross-linking of anionic HA with cationic zein reversed the zeta potential from 34.8 mV (Zein) to −50.2 mV (HZ) (Figure 1D). In addition, the loading of CUR led to a significant decrease in surface charge, suggesting that it was well encapsulated in the nanoparticles Zein-CUR and HZ-CUR. The high zeta potential of HZ and HZ-CUR supported the good stability of the formulations. The hydrogen bonding, electrostatic and hydrophobic interactions between the zein and polysaccharide (HA) molecules were likely responsible for the formation of the stable complexes in the nanoparticles [27]. These characteristic features were accompanied by a significant increase in the EE and LC of the nanoparticles (HZ-CUR) (Table S1) and partly explained the favorable CUR release behavior during simulated gastrointestinal digestion in our previous study [26].

### 2.2. Synergistic Effect of HZ-CUR in Combination with OXA in CRC Cells

**Figure 2:**
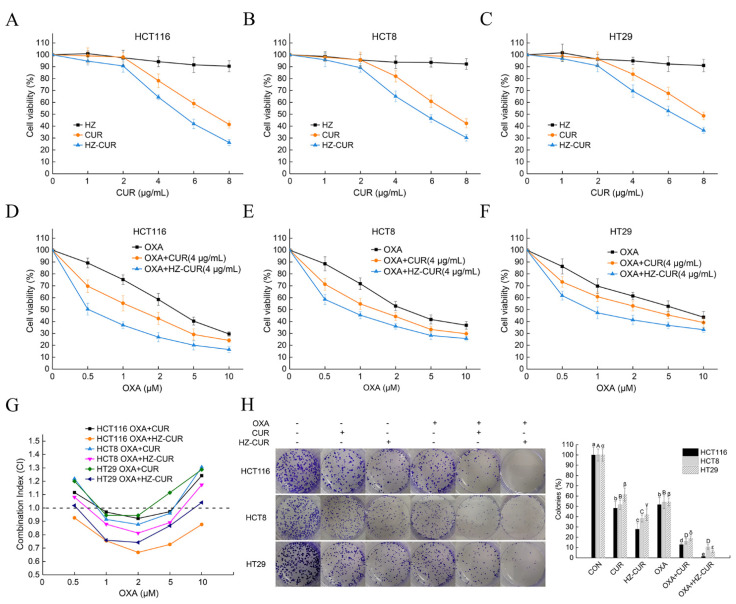
Inhibitory effects of curcumin (CUR) and CUR-encapsulated hyaluronic acid–zein composite nanoparticles (HZ-CUR) alone or in combination with oxaliplatin (OXA) on colorectal cancer (CRC) cell growth. Cells (HCT116, HCT8, and HT29) were treated with the indicated doses of the agents for 48 h, respectively. (**A**–**C**) Effects of CUR, HZ, and HZ-CUR alone on cell viability of CRC cells. (**D**–**F**) Effects of OXA alone or in combination with CUR/HZ-CUR on cell viability of CRC cells. (**G**) Combination Index (CI) values of OXA and CUR/HZ-CUR combination treatments in CRC cells. (**H**) **Left**: images of colony formation assay; **Right**: quantitative analysis of colony formation expressed as percentage relative to control. Values are means ± SD, n = 3. Groups marked with different letters (a, b, c, d, e; A, B, C, D, E or α, β, γ, δ, ε) in the same cell line have significant differences (*p* < 0.05).

The inhibitory effects of CUR and HZ-CUR alone or in combination with OXA on CRC cell growth were evaluated in HCT116, HCT8, and HT29 cells. As shown in Figure 2A–C, CUR and HZ-CUR dose-dependently inhibited the viability of the three CRC cell lines. The inhibitory effect of HZ-CUR was significantly stronger than that of CUR, as evidenced by the 26.0–28.7% lower IC_50_ values in the CRC cell lines (Table S2). Equivalent doses of HZ did not significantly affect CRC cell growth. Similarly, the enhanced cytotoxicity of hydrophobic anticancer drug doxorubicin was achieved with a dual-responsive dendritic polyglycerol sulfate delivery system [28]. CUR and HZ-CUR at 4 μg/mL (<IC_50_) were subsequently selected to assess their potential synergistic effects with OXA (Figure 2D–F). According to their CI, most of the combination treatments with moderate doses of OXA (1–5 μM) exhibited synergistic cytotoxic activity (CI < 1) in the three cell lines. Compared with CUR, HZ-CUR exhibited a stronger synergistic effect with OXA, especially in HCT116 and HT29 cells (Figure 2G). The data from the colony-formation assay also supported the synergistic cytotoxicity effects of both CUR and HZ-CUR with OXA. Similarly, the synergistic effect between OXA was stronger with HZ-CUR than with CUR (Figure 2H). The combinations of 2 µM OXA and 4 μg/mL of CUR or HZ-CUR were found to be significantly better than the other combinations. Hence, they were used in subsequent mechanistic analyses.

### 2.3. OXA Promotes Cellular Uptake of HZ-CUR via Upregulation of CD44

**Figure 3:**
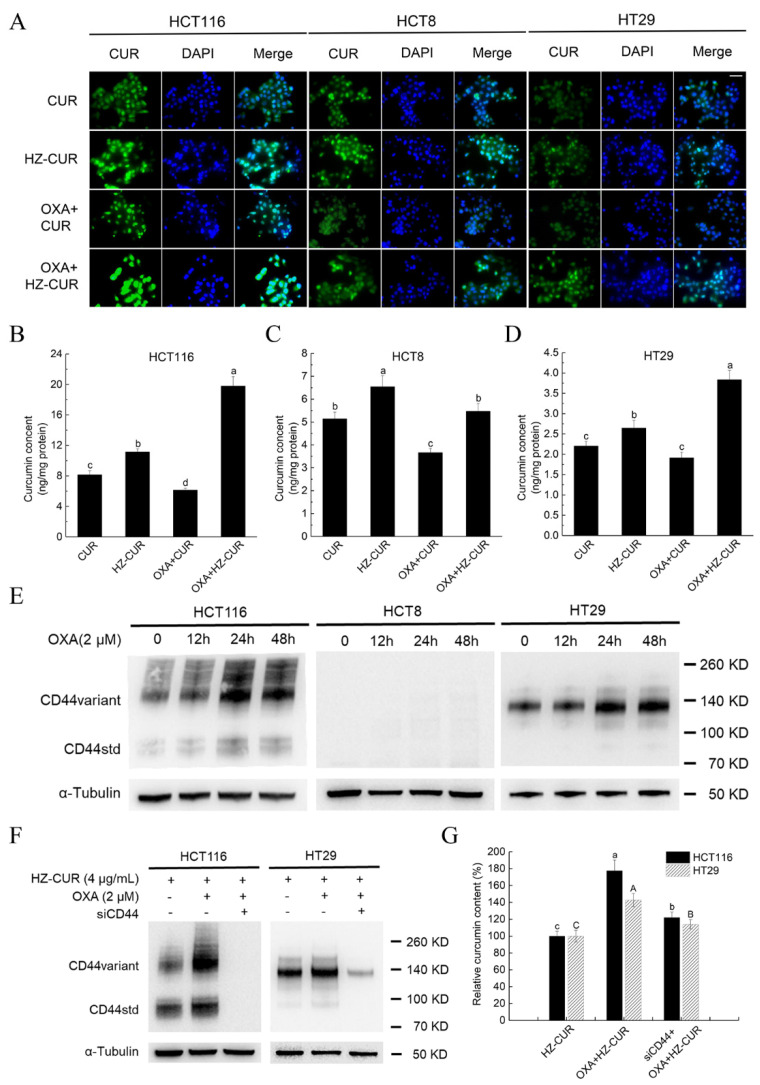
Cellular uptake analysis of CUR and HZ-CUR and Western blot analysis of CD44 expression in CRC cells. Cells were treated with the indicated doses of the agents for 24 h. (**A**) Fluorescence images (400×) of CUR (green). Nuclei were stained with DAPI (blue). Scale bar, 200 μm. (**B**–**D**) CUR contents in HCT116, HCT8, and HT29 cells, respectively. (**E**) Western blot analysis of CD44 expression in CRC cells treated with OXA for 12, 24, and 48 h. α-Tubulin was used as loading control. (**F**) Western blot analysis of CD44 expression and (**G**) relative CUR content in HCT116 and HT29 cells (CD44+/CD44−). Knockdown of CD44 in HCT116 and HT29 cells was conducted with specific siRNAs. Values are means ± SD, n = 3. Groups marked with different letters (a, b, c or A, B, C) in the same cell line have significant differences (*p* < 0.05).

To explore the mechanism of the enhanced synergistic effect of OXA and HZ-CUR relative to OXA and CUR, cellular uptake of these agents alone and in combinations was assayed. Fluorescence imaging and quantitative cellular uptake analysis both showed that compared with free CUR, HZ-CUR treatment resulted in significantly higher CUR contents in the three CRC cell lines after 24 h incubation. Interestingly, cotreatment with OXA inhibited the cellular uptake of free CUR. On the contrary, OXA appeared to have a promoting effect on HZ-CUR uptake, which was supported by the significantly higher cellular contents of CUR in HCT116 and HT29 cells compared to those achieved with HZ-CUR alone (77% and 43%, respectively). This enhancing effect of OXA on cellular uptake of HZ-CUR was not observed in HCT8 cells (Figure 3A–D). These data were consistent with the stronger synergistic cytotoxicity of OXA and HZ-CUR than OXA and CUR against HCT116 and HT29 cells (Figure 2G,H).

CD44 expression has been reported to enhance the chemoresistance of OXA in CRC cells [25]. CD44 is a specific receptor of HA frequently overexpressed in various cancer cells, and this property has been utilized for tumor targeting of HA-coated nanoparticles [24]. To evaluate whether the difference in cellular uptake of HZ-CUR among the three CRC cell lines induced by OXA was related to CD44 targeting, the effect of OXA on CD44 expression in the cell lines was assayed. As shown in Figure 3E, OXA caused upregulation in the expression of CD44 in HCT116 and HT29 cells in a time-dependent manner, whereas CD44 expression was undetectable in HCT8 cells with or without treatment with OXA, which was in large agreement with the data of a previous study [29]. These results indicated that the OXA-induced upregulated expression of CD44 likely contributed to the increased cellular uptake of HZ-CUR in HCT116 and HT29 cells. A previous study also found enhanced specific receptor-mediated cellular uptake of anticancer drug gemcitabine with a PEG-b-poly (carbonate)-derived nanocarrier [30]. To verify the importance of CD44 in the OXA-induced increased cellular uptake of HZ-CUR in HCT116 and HT29 cells, intracellular CUR content in both cell lines was assayed following siRNA-mediated silencing of the expression of CD44 (Figure 3F,G). The results showed that CD44 knockdown significantly (*p* < 0.05) attenuated the OXA-induced increased intracellular CUR content in HCT116 and HT29 cells, suggesting that the increase was largely mediated through OXA-induced upregulation of CD44 expression and that CD44 receptor-mediated endocytosis is an important internalization mechanism of the CRC cells for cellular uptake of the HZ-CUR nanoparticles [31]. CD44 is a transmembrane glycoprotein and has been considered to play an important role in the development of resistance to chemotherapeutic agents such as doxorubicin and OXA [25,32]. It has also been a widely used marker of CRC stem cells whose roles in metastasis, recurrence, and CRC chemoresistance have been amply demonstrated [33,34]. Our data, together with the literature, support that HZ-CUR is a promising agent for targeting OXA-induced CD44 on CRC cells to realize enhanced cellular uptake of CUR and, thus, better chemotherapeutic outcomes from OXA therapy. Apart from the principal cell-surface receptor CD44, RHAMM, ICAM-1, and LYVE-1 have also been reported to be potential cell surface receptors for HA and thus may play a role in binding and promoting its cellular uptake [35,36]. It was also reported that CD44 and RHAMM might have a compensatory role in binding with HA [37]. Therefore, CD44 knockdown did not completely eliminate the OXA-induced increase in cellular uptake of HZ-CUR in HCT116 and HT29 cells. In addition, despite having a negligible expression of CD44, HCT8 was found to have a high expression of ICAM-1 and LYVE-1 [38,39]. Therefore, HZ-CUR may enter HCT8 cells through HA-ICAM-1 and/or HA-LYVE-1 mediated endocytosis. These data and the literature also partly explained the observation that despite negligible expression of CD44 in HCT8 cells, the OXA-HZ-CUR combination still resulted in a higher cellular CUR content (and thus stronger cytotoxicity) compared to the OXA-CUR combination (Figure 3C). Besides these, pathways such as macropinocytosis, clathrin- and caveolin-independent endocytosis may also be involved in the cellular uptake mechanism of HCT8 cells [40]. Nevertheless, the specific mechanism(s) responsible for the higher CUR concentration observed in HCT8 cells compared to HT29 cells treated with free CUR or HZ-CUR requires further studies to characterize.

### 2.4. Anti-Tumor Effect of Plain CUR and HZ-CUR Alone and in Combination with OXA in a Subcutaneous CRC Xenograft Model in Mice

**Figure 4:**
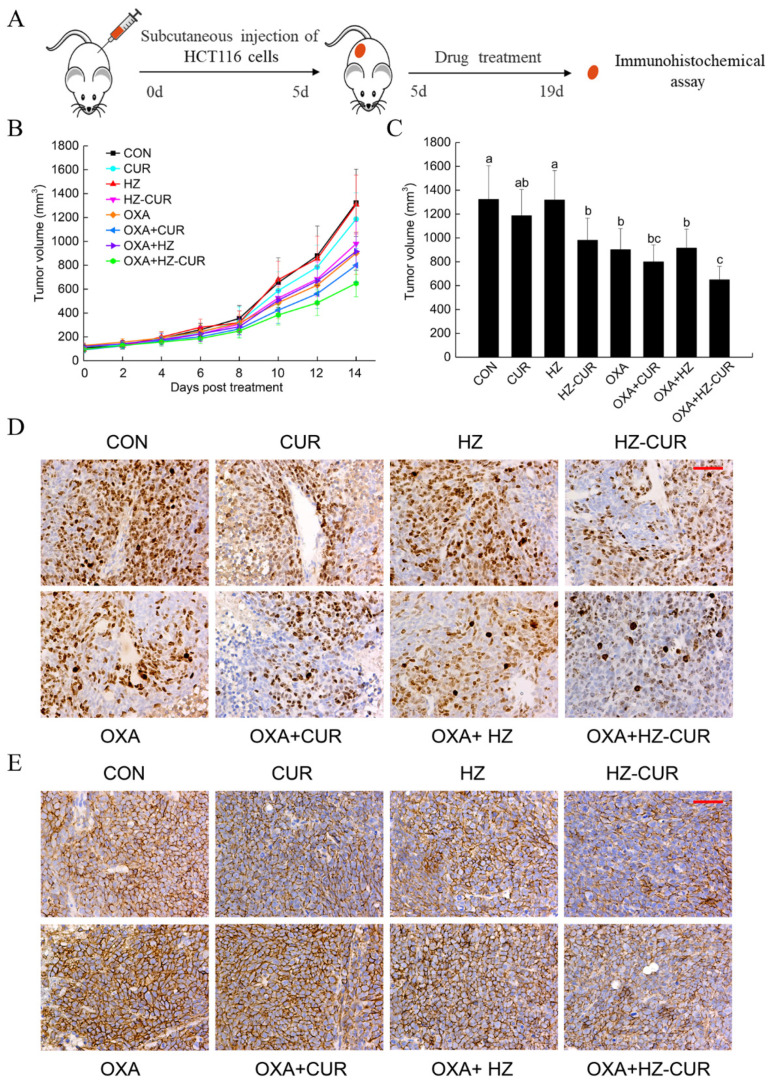
Anti-tumor effect of plain CUR and HZ-CUR alone and in combination with OXA in a subcutaneous xenograft CRC model in mice. (**A**) Experimental scheme. (**B**) Growth curves of tumors in the experimental groups. Values are means ± SD, n = 6. (**C**) Tumor volumes of the experimental groups at the end of the 14-day treatment period. Values are means ± SD, n = 6. Groups marked with different letters (a, b, c, d) have significant differences (*p* < 0.05). Representative immunohistochemical images (200×) of (**D**) Ki67- and (**E**) CD44-stained tumor sections from experimental groups. Scale bar, 50 μm.

Next, a subcutaneous xenograft model of HCT116 cells in BALB/c nude mice was used to verify the differential anticancer effects of the above agents according to the experimental scheme in Figure 4A. Tumor volumes were recorded every other day (Figure 4B,C). At the end of the 14-day treatment period, the tumor volume of the HZ-CUR group was significantly (980.03 mm^3^ vs. 1322.61 mm^3^, *p* < 0.05) lower than that of the vehicle control, while CUR only achieved a moderate (*p* < 0.25) inhibition. Oral administration of blank HZ did not seem to have any effect on tumor growth, whereas a significant inhibitory effect was found in Seok et al.’s study in CT26 xenografts by intravenous injection of blank HZ [41]. The different effects of HZ might be due to the different ratios of zein to HA in the preparation process and the different routes of administration (oral vs. intravenous). In addition, the inhibition rate of the OXA-HZ-CUR combination was significantly (*p* < 0.05) higher than that of OXA alone, while that of the OXA-CUR combination was not (*p* = 0.37). Unexpectedly, whether used alone or in combination with OXA, the anti-tumor effect of HZ-CUR was only moderately (*p* < 0.28 vs. *p* < 0.21) but not significantly stronger than that of CUR. Although our preliminary pharmacokinetic study showed that HZ-CUR possessed better oral bioavailability than CUR [26], the data herein suggest that the low dosage of intragastric HZ-CUR probably did not lead to a sufficient level of CUR in the subcutaneous xenografts. Further studies are warranted to test whether higher oral dosages of the HZ-CUR would generate more significant therapeutic advantages over CUR.

Furthermore, the expression of Ki-67 (a widely used proliferation marker for human tumor cells) and CD44 in the tumor xenografts of the experimental groups was evaluated by immunohistochemical analysis. As shown in Figure 4D, a significantly lower percentage of Ki-67-positive cells was observed in the HZ-CUR and OXA groups compared with control, and the OXA-HZ-CUR combination had a stronger suppressive effect on Ki-67-positive cells than the single agents. In addition, HZ-CUR effectively suppressed the expression of CD44 relative to control, and the OXA-HZ-CUR combination also had a stronger inhibitory effect on CD44 expression than OXA alone (Figure 4E). These data suggested that HZ-CUR had effective inhibitory effects on tumor cell proliferation and CD44 expression, either alone or in combination with OXA.

### 2.5. CRC Model Anti-Tumor Effect of Plain CUR and HZ-CUR Alone and in Combination with OXA in an AOM/DSS-Induced CRC Model in Mice

**Figure 5:**
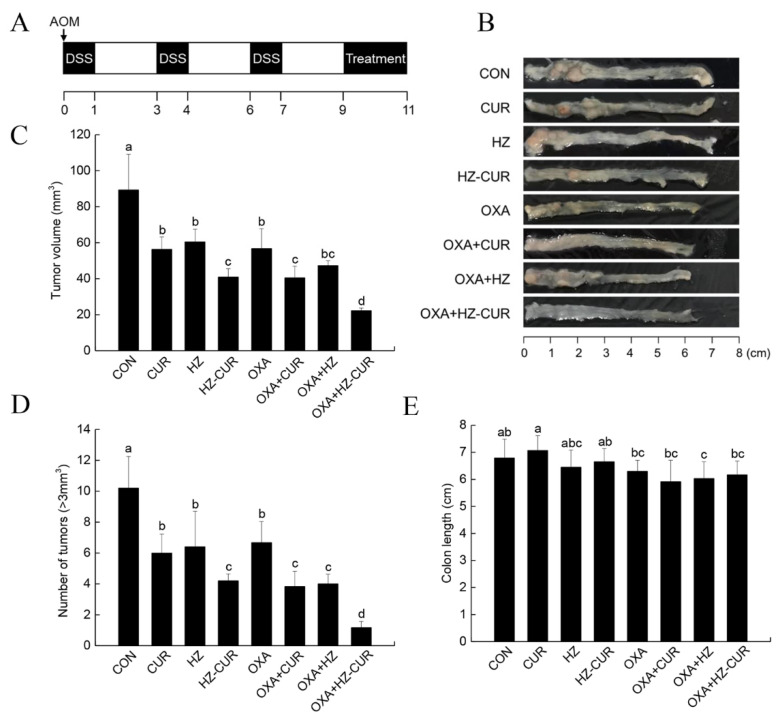
Anti-tumor effect of plain CUR and HZ-CUR alone and in combination with OXA in an AOM/DSS-induced CRC model in mice. (**A**) Experimental scheme. (**B**) Representative colon images of the experimental groups. (**C**) Tumor volume, (**D**) number of tumors (>3 mm^3^), and (**E**) colon length. Values are means ± SD, n = 6. Groups marked with different letters (a, b, c, d) have significant differences (*p* < 0.05).

The above data led us the further assess the anticancer potential of oral HZ-CUR in an AOM/DSS-induced CRC model. The establishment of the model and treatment scheme is outlined in Figure 5A. At the end of the 11-week treatment period, the colons of mice were collected for further analysis. Figure 5B exhibits the representative colon images of the experimental groups. Different from the results in the subcutaneous xenograft model, CUR and HZ both significantly (*p* < 0.05) inhibited tumor growth by 37% and 32%, respectively, when compared with control. HZ-CUR suppressed tumor growth by 54%, which was stronger than the effect of CUR, OXA, and HZ. The inhibition rates of the combination treatments OXA-CUR and OXA-HZ-CUR were 29% and 61% higher than that of OXA alone, respectively, highlighting a significantly stronger synergistic effect between OXA and HZ-CUR than between OXA and CUR (Figure 5C). The inhibitory effects of the agents on the number of tumors (>3 mm^3^) were largely consistent with that of tumor volume (Figure 5D). The colon length was slightly decreased by OXA but not affected by HZ-CUR (Figure 5B,E), suggesting that the oral dosage of HZ-CUR was well tolerable. 

**Figure 6:**
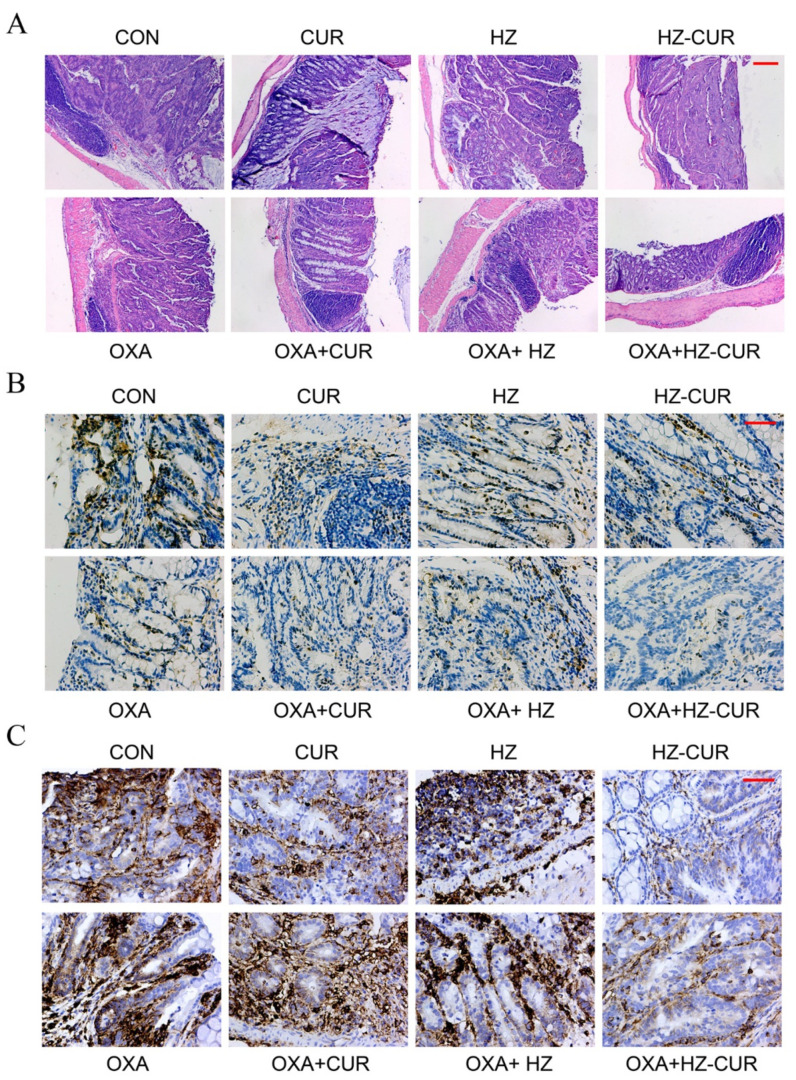
Histopathological and immunohistochemical analysis of colonic tissue sections from mice of the in situ CRC model. (**A**) Representative histological images of hematoxylin- and eosin-stained (40×) colonic tissue sections from the experimental groups. Scale bar, 200 μm. Representative immunohistochemical images (200×) of (**B**) Ki67- and (**C**) CD44-stained colonic tissue sections from experimental groups. Scale bar, 50 μm.

In addition, H&E staining was used to assess the histological structure of colon tissue in each group (Figure 6A). Both CUR and HZ-CUR offered a certain degree of protection against AOM/DSS-induced necrosis of the colonic crypt and goblet cells, and HZ-CUR appeared to have a better effect than CUR. More significant protection of the colonic crypt against tumor progression was observed in the OXA-HZ-CUR combination group. Immunohistochemical staining of the colonic tissue sections showed a significantly lower percentage of Ki-67-positive cells in the HZ-CUR and CUR groups compared with control, and the suppressive effect of HZ-CUR was stronger than that of CUR. Moreover, the OXA-HZ-CUR combination had a stronger suppressive effect on Ki-67-positive cells than the OXA-CUR combination (Figure 6B). These data suggested that HZ-CUR had a stronger inhibitory effect on tumor cell proliferation than CUR, either alone or in combination with OXA. Similar to the trend for Ki-67 expression, both HZ-CUR and CUR effectively suppressed the expression of CD44 relative to control, and the effect of HZ-CUR was much stronger than that of CUR (Figure 6C). Our results are consistent with a previous report by Fan X et al. that CUR could downregulate CD44 expression and decrease the proportion of CD44-positive CRC cells [42]. The lower percentage of CD44-positive cells in the HZ-CUR than the CUR group could be attributed to the enhanced colonic delivery/accumulation of CUR achieved with the former than with the latter agent. This significantly stronger inhibitory effect of HZ-CUR than CUR on CD44 expression was also evident in the combination groups (OXA-HZ-CUR vs. OXA-CUR). CD44 has been a widely used marker for cancer stem cells, and it has been demonstrated to be involved in colonization, invasion, metastasis, and chemoresistance of CRC. Clinical analyses showed that CD44 overexpression could be used to predict poor differentiation and has been considered to be an adverse prognostic factor for CRC patients [43,44,45]. Hayashi H et al. reported that CD44 expression enhanced the chemoresistance of CRC cells to OXA [25]. The strong inhibitory effect of the OXA-HZ-CUR combination on CD44 expression may improve the chemosensitivity of CRC cells and thus the therapeutic efficacy of OXA.

## 3. Materials and Methods

### 3.1. Materials

HA (100–300 kDa, ≥99%) was obtained from Xi’an XABC Biotech Co. Ltd. (Xi’an, China). Zein (Z3625) and OXA (O9512) were purchased from Sigma-Aldrich (St. Louis, MO, USA). CUR was from Tianjin Guangfu Fine Chemical Research Institute (Tianjin, China). Three CRC cell lines (HCT116, HCT8, and HT29) were purchased from the National Infrastructure of Cell Line Resource (Beijing, China).

### 3.2. Preparation of Composite Nanoparticles

HZ-CUR was prepared with an antisolvent coprecipitation method based on a previous study with some modifications [46]. Briefly, free CUR (10 mg) and zein (100 mg) were dissolved in 80% ethanol (*v*/*v*, 10 mL) and then slowly injected into 0.04% HA solution (*m*/*v*, 50 mL) with stirring (600 rpm, 20 min). The solution was concentrated by rotary evaporation (45 °C, −0.1 MPa) to remove the ethanol. This was followed by centrifugation (3000 rpm, 10 min) to remove insoluble particles. Blank HA–zein composite nanoparticles (HZ) were prepared with the same protocol.

### 3.3. Characterization of Composite Nanoparticles

Zeta potential, polydispersity index (PDI), and particle size of the composite nanoparticles were measured with a dynamic light scattering (DLS) particle analyzer (NanoBrook 90Plus, Brookhaven, USA). CUR in composite nanoparticles was extracted with 80% ethanol (*v*/*v*) and quantified at 426 nm in a microplate reader (SpectraMaxTM i3X, Molecular Devices, Silicon Valley, USA). Loading capacity (LC) and encapsulation efficiency (EE) of CUR-encapsulated nanoparticles were calculated using the following equations:

LC = encapsulated CUR (g)/total mass of nanoparticles (g) × 100%.

EE = encapsulated CUR (g)/total CUR (g) × 100%.

### 3.4. Cell Viability Assay

Cytotoxicity of free CUR, HZ-CUR, OXA, and their combinations against CRC cells was evaluated by Cell Counting Kit-8 (CCK-8) assay (CK-04, Dojindo, Kumamoto, Japan). Briefly, 5 × 10^3^ cells were seeded into each well of a 96-well plate and allowed to grow for 24 h. After 48 h treatment with the above agents, the cells were incubated with CCK-8 solution for 1 h and then measured at 450 nm in a microplate reader. IC_50_ values of the different treatments were analyzed by the logistic regression model in SPSS software, Version 19.0. Potential synergistic, additive, or antagonistic effects of the combination treatments were assessed using the method of Chou and Talalay [47]. Combination indices (CI) were calculated using Calculsyn software (Biosoft, Cambridge, UK). CI < 1, CI = 1, and CI > 1 indicate synergistic, additive, and antagonistic effects, respectively.

### 3.5. Colony-Formation Assay 

Cells were seeded into 6-well plates at a density of 1 × 10^3^ cells per well and allowed to grow for 24 h. After 48 h treatment with free CUR, HZ-CUR, OXA, and their combinations, the agents were removed, and the cells were cultured for colony formation. Following fixing with methanol, the colonies were stained with 0.1% crystal violet and then counted to obtain an average sum.

### 3.6. Fluorescence Imaging Assay

Cells were seeded in a chambered coverslip (80426, Ibidi, Martin reder, Germany) at a density of 1 × 10^5^ cells per well. After incubation with the above agents for 24 h, the cells were fixed with 4% paraformaldehyde, stained with 0.1 μg/mL DAPI in the dark, and then observed and imaged with an inverted fluorescence microscope (Axio Vert.A1, Carl Zeiss, Oberkochen, Germany).

### 3.7. UPLC-MS Analysis of CUR

CUR concentration in the samples was determined by UPLC-MS (Waters, Milford, MA, USA) with a BEH reversed-phase C18 column (1.7 μm, 50 × 2.1 mm, Waters). A binary mobile phase (solvent A: acetonitrile; solvent B: water with 0.1% formic acid) was used with the following elution program: 0–6 min, 40–100% A; 6–8 min, 100–40% A. The flow rate was set as 0.3 mL/min, and the injection volume was 1 μL. MS/MS data acquisition was performed under positive electrospray ionization mode. Multiple reaction monitoring mode was employed to monitor CUR with precursor-to-product ion transition of *m*/*z* 369.28/145.13 and *m*/*z* 369.28/177.12. The operating conditions were capillary voltage 3.0 Kv, cone voltage 20 V, desolvation gas flow 600 L/h, and desolvation temperature 400 °C.

### 3.8. Quantitative Cellular Uptake Analysis

Cells were seeded into 6-well plates at a density of 5 × 10^5^ cells per well and allowed to grow for 24 h. After incubation with the above agents for 24 h, the cells were harvested and lysed with RIPA buffer (R0010, Solarbio, Beijing, China) containing 1 mM phenylmethanesulfonyl fluoride (P0100, Solarbio, Beijing, China). The protein concentration of the lysate was measured by using a BCA Protein Assay kit (T9300A, TaKaRa, Otsu, Japan). For quantification of cellular CUR, the lysate was extracted with acetonitrile and centrifuged, and the supernatant was analyzed by UPLC-MS. The content of CUR in cells was normalized to total protein content.

### 3.9. Western Blot Analysis

Cells were treated with OXA or vehicle for 12, 24, or 48 h and then lysed in RIPA buffer-containing protease inhibitor cocktail (04693116001, Roche, Basel, Switzerland). Proteins were resolved by SDS-PAGE, transferred to PVDF membranes (162–0177, Bio-Rad, Berkeley, CA, USA), and probed against CD44, 37259, 1:1000 Cell Signaling Technology, Beverly, CA, USA). Specific bands were visualized using a chemiluminescence kit (1856135, Thermo Fisher Scientific, Waltham, MA, USA). α-Tubulin (ab15246, 1:1500, Abcam, Cambridge, UK) was used as loading control.

### 3.10. RNA Interference Assay

Specific siRNAs targeting CD44 were transfected into CRC cell lines using Lipofectamine™ 2000 Transfection Reagent (Invitrogen, Carlsbad, CA, USA) according to the manufacturer’s instructions. Before transfection, cells were plated in a growth medium without antibiotics such that they would be 30–50% confluent at the time of transfection. Briefly, 100 pmol of siRNA was diluted in 100 µL of Gibco+™ Opti-MEM™ I reduced serum medium. Ten microliters of Lipofectamine 2000 were mixed with 100 µL of Opti-MEM medium and incubated for 5 min at room temperature. The diluted siRNA was mixed with the diluted Lipofectamine, and treatments with drugs or vehicle were conducted after incubation for 48 h.

### 3.11. Animal Studies

BALB/c nude mice and BALB/c mice (female, six-week-old) were purchased from Guangdong Medical Laboratory Animal Center (Foshan, China). The mice were kept under pathogen-free conditions with a 12/12 h light/dark cycle and allowed to acclimatize for 7 days before experimentation.

Anti-tumor study in a subcutaneous xenograft model. HCT116 cells were suspended in a serum-free medium containing 50% Matrigel and injected subcutaneously into the right flanks of BALB/c nude mice at a density of 1 × 10^6^ cells per mouse. Five days after implantation, the mice were randomly divided into eight groups (six mice per group): control, CUR, HZ, HZ-CUR, OXA, OXA plus CUR, OXA plus HZ, and OXA plus HZ-CUR. Intragastric administration of CUR (dissolved in 0.5% sodium carboxymethylcellulose, 6 mg/kg), HZ-CUR (equivalent to 6 mg/kg CUR), and HZ was performed daily. Intraperitoneal injection of OXA (dissolved in 5% glucose solution, 7.5 mg/kg) was performed twice a week. Water and 5% glucose solution were given to the mice as vehicle controls of the corresponding treatment groups, respectively. Tumor volumes and body weights were measured three times a week. Tumor volume was calculated according to the formula: tumor volume = (tumor width)^2^ × (tumor length)/2. After two weeks of treatment, the mice were sacrificed by cervical dislocation, and the tumors were excised for further analysis.

Anti-tumor study in an in situ CRC model. BALB/c mice were intraperitoneally injected with a single dose (10 mg/kg) of azoxymethane (AOM) (A5486, Sigma-Aldrich, St. Louis, MO, USA), followed by three cycles of oral administration of 2% dextran sodium sulfate (DSS) (0216011090, MP Biomedicals, Santa Ana, CA, USA) for one week and normal drinking water for two weeks. Subsequently, the mice were randomly divided into eight groups (six mice per group), which were subjected to the treatments as described in the subcutaneous xenograft model. At the end of the treatment, the mice were sacrificed by cervical dislocation, and the colons were resected, longitudinally dissected, and washed. The number and volume of tumors in each colon were documented. Tumor volume was calculated according to the formula: tumor volume = (tumor width)^2^ × (tumor length)/2.

### 3.12. Immunohistochemical (IHC) and Histopathological Analysis

Tumors and colon tissues of the mice were fixed with 4% paraformaldehyde, embedded in paraffin, and cut into 5 μm sections. Immunohistochemical staining of the tissue sections was conducted as previously described [48]. Antibodies against ki67 (ab279653, 1:1000, Abcam, Cambridge, UK) and CD44 (37259, 1:100, Cell Signaling Technology, Beverly, MA, USA) were used. Additionally, the colon tissues were stained with hematoxylin and eosin (H&E) for histological analysis. Multiple fields of each tissue section were randomly selected for IHC (200×) and histopathological (40×) analyses.

### 3.13. Statistical Analysis

The data are presented as means ± SDs. One-way ANOVA and Duncan test were used to assess statistical significance among groups. *p* < 0.05 was considered to be statistically significant. SPSS software was used for the analyses.

## 4. Conclusions

The present study has demonstrated that HZ-CUR was more effective than CUR against CRC cells growth, especially when combined with OXA. The enhanced anti-CRC effect of HZ-CUR might be attributed to its increased cellular uptake when compared with free CUR. The increased cellular uptake could be further promoted when combined with OXA through OXA-induced upregulation of CD44 expression. The effects were further validated in a subcutaneous CRC xenograft model and an in situ CRC model in mice. Altogether, the findings support the great potential of HZ-CUR as an efficacious oral adjuvant for OXA-based CRC chemotherapies. Further studies are warranted to better understand the therapeutic efficacy of the HZ-CUR and OXA combination in different ratios and dosing schemes.

## Data Availability

Data related to the present study are available from the corresponding author upon reasonable request.

## Supplementary Materials

The following supporting information can be downloaded online. Table S1: Encapsulation efficiency (EE) and loading capacity (LC) of CUR-loaded composite nanoparticles; Table S2: IC_50_ values of CUR and HZ-CUR in HCT116, HCT8, and HT29 cells.
