# Supplementary material for: Hyaluronic Acid–Zein Core-Shell Nanoparticles Improve the Anticancer Effect of Curcumin Alone or in Combination with Oxaliplatin against Colorectal Cancer via CD44-Mediated Cellular Uptake
Source: Molecules. 2022 Feb 23;27(5):1498. doi: 10.3390/molecules27051498 (PMC8911772; doi:10.3390/molecules27051498)
Supplement: Supplementary file 1 [file molecules-27-01498-s001.zip › molecules-1593610-supplementary.pdf]

Supplementary Material

Supplementary Table S1 Encapsulation efficiency (EE) and loading capacity (LC) of CUR-loaded composite nanoparticles.

	EE (%)	LC (%)
Zein-CUR	76.49 ± 3.02	6.95 ± 0.27
HZ-CUR	97.12 ± 1.86 *	7.47 ± 0.14 *

Values are means ± SD, n = 3, $^{*}p < 0.05$ relative to Zein-CUR .

Table S2 IC_{50} values of CUR and HZ-CUR in HCT116, HCT8, and HT29 cells.

IC_{50} (μg/mL)	HCT116	HCT8	HT29
CUR	6.79 ± 0.83	7.45 ± 0.87	8.52 ± 0.93
HZ-CUR	5.03 ± 0.70 *	5.31 ± 0.73 *	6.3 ± 0.80 *

Values are means ± SD, n = 3, $^{*}p < 0.05$ relative to CUR.
